# Isoform-Specific Compensation of Cyclooxygenase (*Ptgs*) Genes during Implantation and Late-Stage Pregnancy

**DOI:** 10.1038/s41598-018-30636-x

**Published:** 2018-08-14

**Authors:** Xinzhi Li, Laurel L. Ballantyne, Mackenzie C. Crawford, Garret A. FitzGerald, Colin D. Funk

**Affiliations:** 1 Department of Biomedical and Molecular Sciences, Queen’s University, Kingston, ON Canada; 2 Institute for Translational Medicine and Therapeutics, Perelman School of Medicine, University of Pennsylvania, Philadelphia, PA USA

## Abstract

The participation of cyclooxygenase (COX) in embryo implantation and parturition has been studied extensively. However, the distinct role of the two COX isoforms in these processes still remains unclear. Using three characterized mouse lines where the *Ptgs1* and *Ptgs*2 genes substitute for one another, this study focused on the reproductive significance of their distinct roles and potential biological substitution. In both non-gravid and gravid uteri, the knock-in COX-2 is expressed constitutively, whereas the knock-in COX-1 is slightly induced in early implantation. The delayed onset of parturition previously found in COX-1 null mice was corrected by COX-2 exchange in COX-2>COX-1 mice, with normal term pregnancy, gestation length and litter size. In contrast, loss of native COX-2 in COX-1>COX-2 mice resulted in severely impaired reproductive functions. Knock-in COX-1 failed to substitute for the loss of COX-2 in COX-1>COX-2 mice during implantation, indicating that COX-1 may be replaced by COX-2, but not vice versa. A panel of prostaglandins detected in uterus and ovary demonstrates that prostaglandin biosynthesis preferentially depends on native COX-1, but not COX-2. More interestingly, preferential compensations by the COX isoforms were sustained despite weak dependency on their role in prostaglandin biosynthesis in the uterus and ovary.

## Introduction

Prostaglandin (PG) H synthase exists as two isoforms, also known as cyclooxygenases (COX-1 and -2) and encoded by *Ptgs1* and *2* genes, respectively. COX-2 mRNA (4.1 kb) has an AU-rich 3′-untranslated region which is responsible for a faster mRNA turnover compared with that of the major COX-1 mRNA (2.8 kb). COX-1 and COX-2 are homodimeric, heme-containing, glycosylated proteins with two catalytic sites: cyclooxygenase and peroxidase^1^. At the amino acid level, COX-1 and COX-2 are 60% identical, including similar signal peptides, epidermal growth factor-like domains, membrane-binding domains, and catalytic domains. The two enzymes are also similar in terms of the crystal structure with one principal difference being an extra “side pocket” only found in COX-2, but not COX-1, which allows more space in the active site for substrates^2–4^. Both isoforms are anchored in the endoplasmic reticulum and nuclear membrane^5^, but COX-2 also resides in the Golgi apparatus^6^. They differ markedly in their tissue expression patterns and responses to regulatory stimuli. In simplistic terms, COX-1 is largely constitutively expressed, whereas COX-2 is often inducible by cytokines, growth factors, and hormones^7^. Both COX isoforms initiate the biosynthesis of a family of bioactive lipid mediators, known as PGs (e.g. PGE_2_, PGD_2_, PGF_2α_, PGI_2_) and thromboxane (TxA_2_), in a cell-type restricted fashion^8^. PGs are released from cells and exert a variety of actions, which are mediated by G protein-coupled receptors expressed on neighboring target cells^9^.

Although the two COX isoforms catalyze the same PG biosynthetic reaction, their expression in the reproductive system is spatiotemporally and cell-specifically regulated. Both COX-1 and COX-2 are expressed in epithelial cells of human endometrium and in surface epithelial cells of the ovary, with the stroma staining positive only for COX-2^10^. *Ptgs1* is developmentally regulated in uterine epithelial cells during peri-implantation, whereas *Ptgs2* is highly expressed in luminal epithelium and stromal cells on day 1 of pregnancy^11^. Rapid, but transient, induction of COX-2, but not COX-1, in granulosa cells is observed during ovulation^12,13^. COX-2 present in uterine epithelium, stroma, and the necks of endometrial glands at sites of implantation, appears to be induced in the endometrium by the embryo^14^. *Ptgs2* gene disruption leads to multiple reproductive failures^15,16^, whereas *Ptgs1*-deficient mice have normal fertility, except for parturition defects^17,18^. A series of elegant studies also established isoform-specific roles for PG production at different stages of pregnancy^16,19,20^. Induced COX-2 expression within the follicle^12^ can modulate ovulation through the biosynthesis of PGE_2_^21,22^. COX-1-derived PGF_2α_ is responsible for corpus luteum regression and triggers parturition^23–25^. COX-2-derived prostacyclin (PGI_2_) may be the eicosanoid that mediates embryo implantation and uterine decidualization^16,26^. PGE_2_ production and activation through its E prostanoid (EP) receptor 2 is also required for embryo implantation^27,28^.

Despite these findings, the specific functions of each COX isoform in reproduction still remain unclear or even controversial. For example, *Ptgs2* null mice generated on a mixed C57BL/6 × 129/Sv genetic background were largely infertile^15^. Lim *et al*.^16^ revealed that disruption of COX-2 in this cohort of mice caused failure in multiple female reproductive processes. However, other researchers challenged these findings because they failed to observe any substantial effect on embryo implantation frequencies in *Ptgs2* null mice^29^. In addition, *Ptgs*-associated reproductive functions are conditioned by the genetic background. Female *Ptgs2*^−/−^ mice generated on a mixed C57BL/6 × 129/Sv genetic background are infertile^15,16^, without any compensation from *Ptgs1* gene. However, *Ptgs2* null mice, on a CD-1 background, have dramatically improved female fertility due to a compensatory up-regulation of *Ptgs1*^30^.

Our previously established COX-1>COX-2 mouse strain showed that insertion of COX-1 under control of the COX-2 (*Ptgs2*) promoter could partially rescue the impact of COX-2 deletion on reproductive function^31^. Most recently we successfully generated the COX-2>COX-1 mouse strain using gene targeting. We have then “flipped” the *Ptgs* genes to create COX “*Reversa”* mice where one isoform is replaced by the other via cross-breeding of COX-1>COX-2 and COX-2>COX-1 mice^32,33^ (Table 1**)**. Studies of these mice revealed COX isoform-specific compensatory functions and variable degrees of interchangeability with respect to the capacity for prostaglandin formation in macrophages^32,33^ and kidneys^34^. Here, we took a further step to define the distinct roles of the two COX enzymes in embryo implantation and parturition by examining ovarian and uterine function using COX isoform substitution.

**Table 1:** Mouse strains and their COX isoform expression patterns.

Genotype	Mouse strain	Abbreviation	Native COX-1	Native COX-2	Knockin COX-1	Knockin COX-2
*Wild type*	Wild type	WT	constitutive	inducible	N/A	N/A
*Ptgs2*>*Ptgs1*	COX-2>COX-1	COX-2>1	disrupted	inducible	N/A	constitutive
*Ptgs1*>*Ptgs2*	COX-1>COX-2	COX-1>2	constitutive	disrupted	inducible	N/A
*Reversa*	Reversa	Reversa	disrupted	disrupted	inducible	constitutive

The COX isoform expression patterns are summarized in simplistic terms, where native COX-1 is constitutively expressed and responsible for basal prostaglandin synthesis, whereas native COX-2 is inducible and important in various induced settings. There are notable exceptions to this over-simplification. N/A, not applicable. For more details, please see refs^31–34^.

## Results

### COX exchange does not alter estrous cyclicity

**Figure 1:**
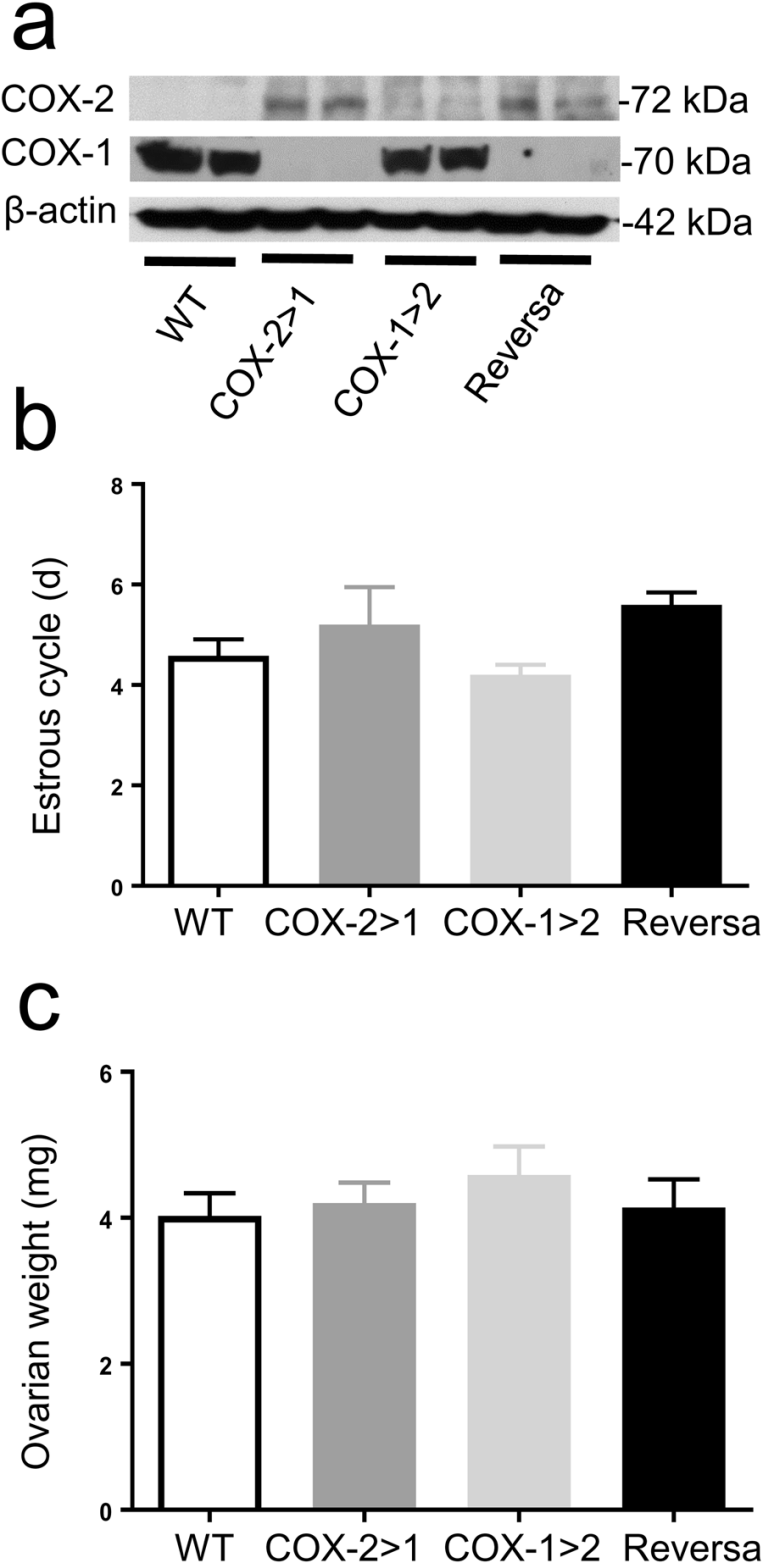
*Ptgs* gene exchange and estrous cyclicity. (**a**) Western blot analysis of COX-1 and COX-2 expression in non-pregnant female uterus, showing knockout of COX-1 and knock-in of COX-2. Images are representative of 3 separate experiments. (**b**) Average estrous cycle length of four groups of mice (10–12 weeks old). At least two cycles were generally captured for each mouse and then averaged to get a cycle length of each mouse. Data are presented as mean ± SEM, n = 5–6. (**c**) Average weight of both ovaries from non-gravid mice (10–12 weeks old). Data are presented as mean ± SEM, n = 7.

Western blot analysis shows that, in non-pregnant mice, COX-1 (70 kDa) is completely knocked-out in all COX-2>COX-1 and Reversa uteri, as expected. However, COX-2 (72 kDa) expression is detectable in both COX-2>COX-1 and Reversa mice under basal conditions, but is absent in WT mice. *Ptgs1-*driven COX-2 expression is no longer “inducible”, but “constitutive”; thus, *Ptgs2*-driven COX-1 expression in Reversa mice was barely observed in the absence of stimulus (Fig. 1a). All four genotypes displayed regular cycling and cytology patterns. There are no significant differences in average cycle length between WT (4.6 ± 0.3 d, n = 6), COX-2>COX-1 (5.2 ± 0.7 d, n = 5), COX-1>COX-2 (4.2 ± 0.2 d, n = 5) or Reversa (5.6 ± 0.2 d, n = 5) mice (Fig. 1b). Moreover, the ovarian weights of COX-2>COX-1, COX-1>COX-2, and Reversa mice are similar to those of WT mice (Fig. 1c). These findings were consistent with the previous evidence that there was no apparent difference in estrous cyclicity between wild-type, *Ptgs1*-deficient, and *Ptgs2*-deficient mice^20^.

### COX isoforms differentially compensate for term pregnancy

To explore whether *Ptgs* gene exchange exhibited differential reproductive phenotypes, we first investigated full-term pregnancy, with a focus on the percentage of mice coming to term, the length of gestation and live litter size. Female homozygous COX-2>COX-1, COX-1>COX-2, and Reversa mice expressed differences in their reproductive capacities (Table 2). On a C57BL/6 genetic background, we failed to generate any COX-1>COX-2 mice from numerous homozygous parent matings. Only one homozygous mother of ten (10%) was successfully brought to term (Table 2), but she did not deliver live pups. This severe phenotype was similar to that observed in COX-2-deficient mice that expressed multiple reproductive defects^16^. These defects were not caused by infertile COX-1>COX-2 males, because homozygous COX-1>COX-2 females (n = 6) failed to reach term-pregnancy when mated with fertile WT males. However, homozygous COX-1>COX-2 males (n = 8) demonstrated normal mating capacity when paired with fertile WT females (Table 2).

**Table 2:** Isoform-specific compensatory functions for term pregnancy.

Maternal Genotype	Paternal Genotype	Sperm-positive	Term pregnancy	Gestation Length (d)	Litter size
**First set**					
WT	WT	10	10 (100%)	19.3 ± 0.1	6.3 ± 0.8
COX-2>COX-1	WT	7	7 (100%)	19.8 ± 0.2	6.4 ± 1.0
COX-1>COX-2	WT	6	0 (0%)	—	–
Reversa	WT	8	5 (62%)	21.0 ± 0.3^*^	1.4 ± 1.0^**^
COX-2>COX-1	COX-2>COX-1	9	9 (100%)	19.5 ± 0.2	6.3 ± 0.5
COX-1>COX-2	COX-1>COX-2	10	1 (10%)	—	—
WT	COX-1>COX-2	8	7 (88%)	19.4 ± 0.3	6.9 ± 0.8
Reversa	Reversa	14	11 (78%)	20.5 ± 0.3^*^	2.1 ± 0.5^**^
**Second set**					
WT	WT	10	—	—	7.0 ± 0.8
COX-2>COX-1 (HM)	COX-2>COX-1 (HM)	10	—	—	6.5 ± 0.5
COX-1>COX-2 (HT)	COX-1>COX-2 (HM)	13	—	—	4.4 ± 0.6^*^
Reversa^§^ (HM/HT)	Reversa (HM)	12	—	—	7.7 ± 0.4

In the first set, female WT, COX-2>COX-1, COX-1>COX-2, and Reversa mice (8–10 weeks old) were bred with fertile WT males or with the same genotypes. Copulation plug-positive mice were observed for delivery at term, and the litter size was monitored closely. Bred with COX-1>COX-2 or WT mice, COX-1>COX-2 females failed to generate any live pups. Delayed parturition occurred in Reversa mice, and their litter size was significantly smaller,^*^P < 0.05,^**^P < 0.01 vs WT or COX-2>COX-1. WT, wild type.

In the second set, the term pregnancy and gestation length were not monitored. HM, homozygous; HT, heterozygous. Reversa^§^ has two copies of *Ptgs2*>*Ptgs1* (HM), but one copy of *Ptgs1*>*Ptgs2* (HT). ^*^P < 0.05 vs WT.

It has been reported that defects in parturition due to COX-1 disruption cannot be compensated for by unimpaired COX-2 activity alone^18^. In contrast to very few COX-1>COX-2 females (0–10%) capable of sustaining term pregnancy, COX-2>COX-1 females (100%) tested were fertile with a normal litter size, when set up with either COX-2>COX-1 (n = 9) or WT (n = 7) males. Moreover, the gestation length of COX-2>COX-1 is comparable to that of WT mice (Table 2). This indicates that knock-in of COX-2 alone or when it is combined with endogenous COX-2 is capable of restoring the normal onset and progression of term labor, which is delayed by COX-1 disruption^17,18^. Although COX-1 is essential for normal parturition, it is also replaceable, given the presence of *Ptgs1*-driven COX-2 expression. Interestingly, these compensatory functions in COX-2>COX-1 mice are not fully maintained in Reversa mice. They have a markedly reduced litter size compared with WT mice, and a longer gestation period (both *P* < 0.05 compared with WT, Table 2). However, the frequency with which they came to term (~70%) exceeded that in COX-1>COX-2 females. Loss of native COX-2 appears to reduce litter size, and this phenotype is not fully rescued by *Ptgs1*-derived COX-2.

To circumvent these female reproductive defects, we set up mating pairs of heterozygous COX-1>COX-2 females with homozygous males. Female Reversa mice with two copies of *Ptgs2*>*Ptgs1* (homozygous) and one copy of *Ptgs1*>*Ptgs2* (heterozygous) were paired with double homozygous Reversa males (Table 2). We monitored the neonatal pups and found that, with a copy of native *Ptgs2* gene, heterozygous COX-1>COX-2 females (n = 13) were able to sustain partially their reproductive capacity as evaluated by litter size relative to WT mice (4.4 ± 0.6 *vs* 7.0 ± 0.8, *P* < 0.05). The native COX-2 from one copy of *Ptgs2*, when combined with knock-in COX-2 in Reversa mice (n = 12) could completely rescue female reproductive defects (Table 2). These results indicate that female reproductive function is gene-dose dependent for native COX-2, but not completely compensated by *Ptgs2* gene insertion into the *Ptgs1* genetic locus in COX-2>COX-1 mice.

### Differential compensatory functions of COX-2 and COX-1 for implantation

**Figure 2:**
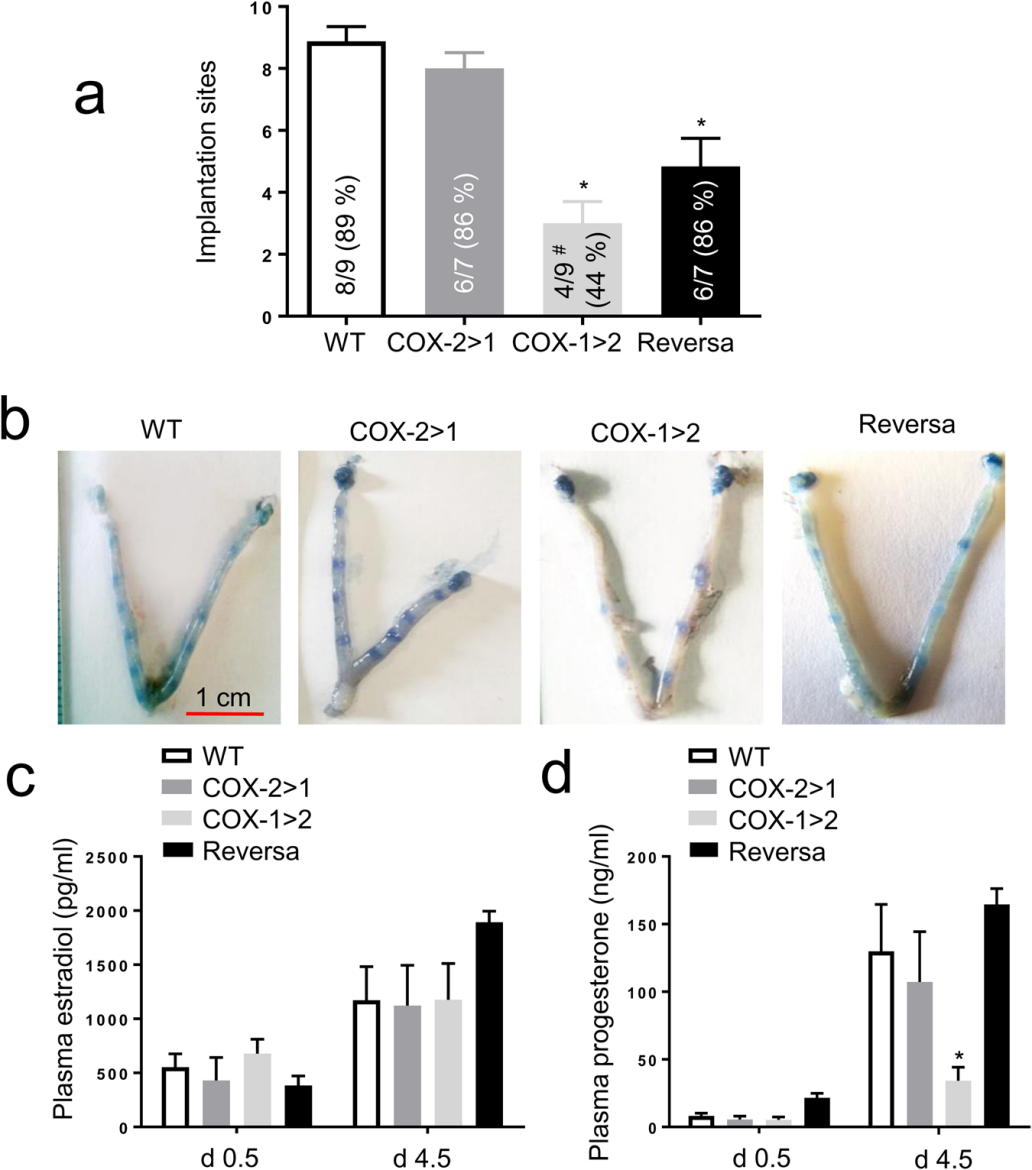
Implantation and plasma sex hormones on gestation day 4.5 (d4.5). (**a**) The number of implantation sites and positive pregnancy rates. A positive pregnancy in implantation experiments is defined as a mouse that displayed at least one visible implantation site on d4.5 of pregnancy. As such, the numbers within the bars indicate the pregnancy (%) that denotes the proportion of mice with implantation sites out of the total mice plugged. Data are presented as mean ± SEM, ^*^P < 0.05 *vs* WT, one-way ANOVA analysis; ^#^*P* < 0.05 *vs* WT, *Chi*-square test. (**b**) Representative photographs of uteri with implantation sites stained with 1% Evan’s blue on d4.5. (**c**) Plasma estradiol and (**d)** progesterone concentration on gestation d0.5 and d4.5, respectively. Blood was collected via submandibular vein from mice of each genotype (n = 4–7) and concentration was determined by sequential competitive immunoassay. Data are presented as mean ± SEM, ^*^P < 0.05 *vs* WT, one-way ANOVA analysis.

We next investigated whether the cause of this failure was aberrant implantation in COX-1>COX-2 mice, and whether COX-2>COX-1 mice restored these functions. In mice, the “window” of implantation is when the uterus becomes receptive on day 4.5 of pregnancy and the implantation-competent blastocyst establishes a reciprocal interaction with the receptive uterus^35^. In this window of implantation, we observed that 6 of 7 (86%) COX-2>COX-1 mice showed distinct implantation sites (8.0 ± 0.5/mouse) on day 4.5 of pregnancy, which was a comparable number of blastocysts implanted in WT mice (89% and 8.9 ± 0.5/mouse). However, only ∼3 implantation sites (3.0 ± 0.7/mouse, *P* < 0.05) were detected in 44% (4/9) of the plug-positive COX-1>COX-2 mice (*P* < 0.05 *vs* WT, *Chi*-square test). In Reversa mice, although the percentage of pregnancies (86%) that may result in live births appeared to be normal, an average number of implantation sites (4.8 ± 0.9/mouse) on day 4.5 was less than that of WT and COX-2>COX-1 mice (*P* < 0.05) (Fig. 2a). This may be caused by the differential spatiotemporal expression of native COX-2 and *Ptgs1*- driven COX-2, since differential expression patterns of native COX-2 and COX-1 have been reported previously^30^. All implantation sites were normally spaced along the uterine horns in WT and the other three strains of mice (Fig. 2b). Histologically, in terms of the relative uterine lumen width, appearance of uterine lumen epithelium, crypt formation and presence or absence of uterine glands, there were no comparable differences across the four genotypes of mice on gestation day 4.5 (data not shown).

Although ≈3 implantation sites (Fig. 2a) were recorded on day 4.5 of pregnancy in COX-1>COX-2 mice, none of them gave rise to live births (Table 2). In Reversa mice, there was only a partial rescue of the implantation defect observed in the presence of the knock-in COX-2, but the litter size at term was still smaller, averaging 2 pups/litter (Table 2). This suggests that blastocysts, which implanted during the normal window, became susceptible to subsequent developmental anomalies, mainly due to lack of native COX-2, leading to few (or no) litters. Similar observations have been made in *Ptgs2*-null mice in which implantation of blastocysts occurs beyond the normal window^30^. Collectively, implantation of blastocysts and live term-pregnancy in Reversa mice suggests that one cause of improved reproductive capacity relative to COX-1>COX-2 mice was the compensation by COX-2, but not vice versa.

There are no significant alterations of basal estradiol levels among the 4 different strains of mice. Timely implantations trigger a rise of plasma estradiol in WT mice and all three mutant strains (Fig. 2c), with concordance across 4 groups. This is in agreement with a previous report showing serum gonadotropin and steroid levels in *Ptgs1*-and *Ptgs2*-deficient mice were unchanged compared with WT mice^20^. Furthermore, this may also explain why all four strains exhibited relatively normal estrous cyclicity^20^. However, the level of progesterone in COX-1>COX-2 mice is much lower than for other three strains on day 4.5 of pregnancy (Fig. 2d). This indicates that loss of COX-2 impairs initiating the conversion of the residual follicle into a corpus luteum that, in turn, produces progesterone to prepare the endometrium for a possible implantation. In Reversa mice, constitutively expressing COX-2, the development of the corpus luteum is rescued, which partially restores blastocyst implantation during the anticipated time course.

### Blastocyst implantation and development of the corpus luteum rely on the presence of COX-2

**Figure 3:**
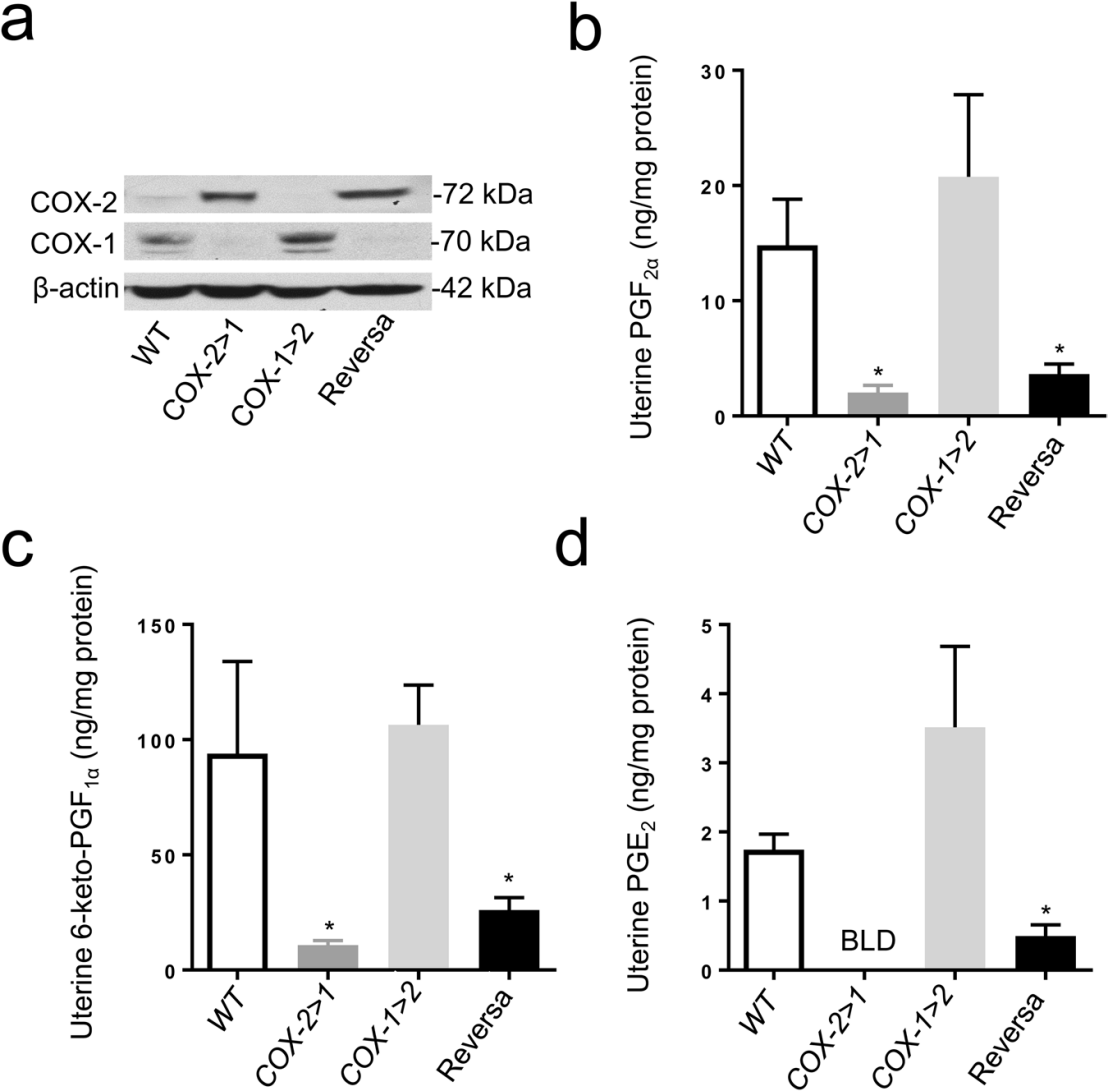
Uterine capacity to generate prostaglandins on gestation day 4.5 (d4.5). (**a**) Western blot analysis of COX-1 and COX-2 expression in uteri on gestation d4.5. Images are representative of 3 separate experiments. (**b**), (**c**), and (**d**) Uterine prostaglandin F_2α_ (PGF_2α_), 6-keto-prostaglandin F_1α_ (6-keto-PGF_1α_), and prostaglandin E_2_ (PGE_2_), respectively. Prostaglandins were extracted, determined by competitive immunoassay, and further normalized with the protein level. Data are presented as mean ± SEM, n = 5–6, ^*^P < 0.05 *vs* WT, one-way ANOVA analysis. BLD, below the limit of detection.

To address which COX isoform and which PGs mediate the process of implantation, we measured the uterine expression of both COX isoforms and the downstream PGs on day 4.5 of pregnancy. A trace of COX-2 expression was observed in WT uterus, possibly due to the induction of COX-2 during early implantation^11,36,37^. Native *Ptgs1*-driven COX-2 was expressed abundantly in both COX-2>COX-1 and Reversa mice, where native COX-1 expression is absent (Fig. 3a). In the present study, COX-1 expression was expected, but not detected in uteri of Reversa mice. However, COX-1>COX-2 uteri expressed a slightly higher amount of COX-1 protein compared with WT counterparts (Fig. 3a), indicating the knock-in *Ptgs1* gene makes a minor contribution to COX-1 protein expression under control of the *Ptgs2* promoter in early implantation. Surprisingly, the knock-in COX-2 protein is not functioning to generate PGF_2α_, 6-keto-PGF_1α_ or PGE_2_ (Fig. 3b–d). These findings are more evident in COX-2>COX-1 mice, where COX-1 is completely replaced by COX-2. Reversa mice can only partially restore the PG-synthesizing function (especially PGE_2_) resulting from the knock-in COX-1. These data are consistent with PGs being primarily generated by uterine COX-1. Moreover, none of these COX-1-derived PGs is indispensable to the process of implantation.

**Figure 4:**
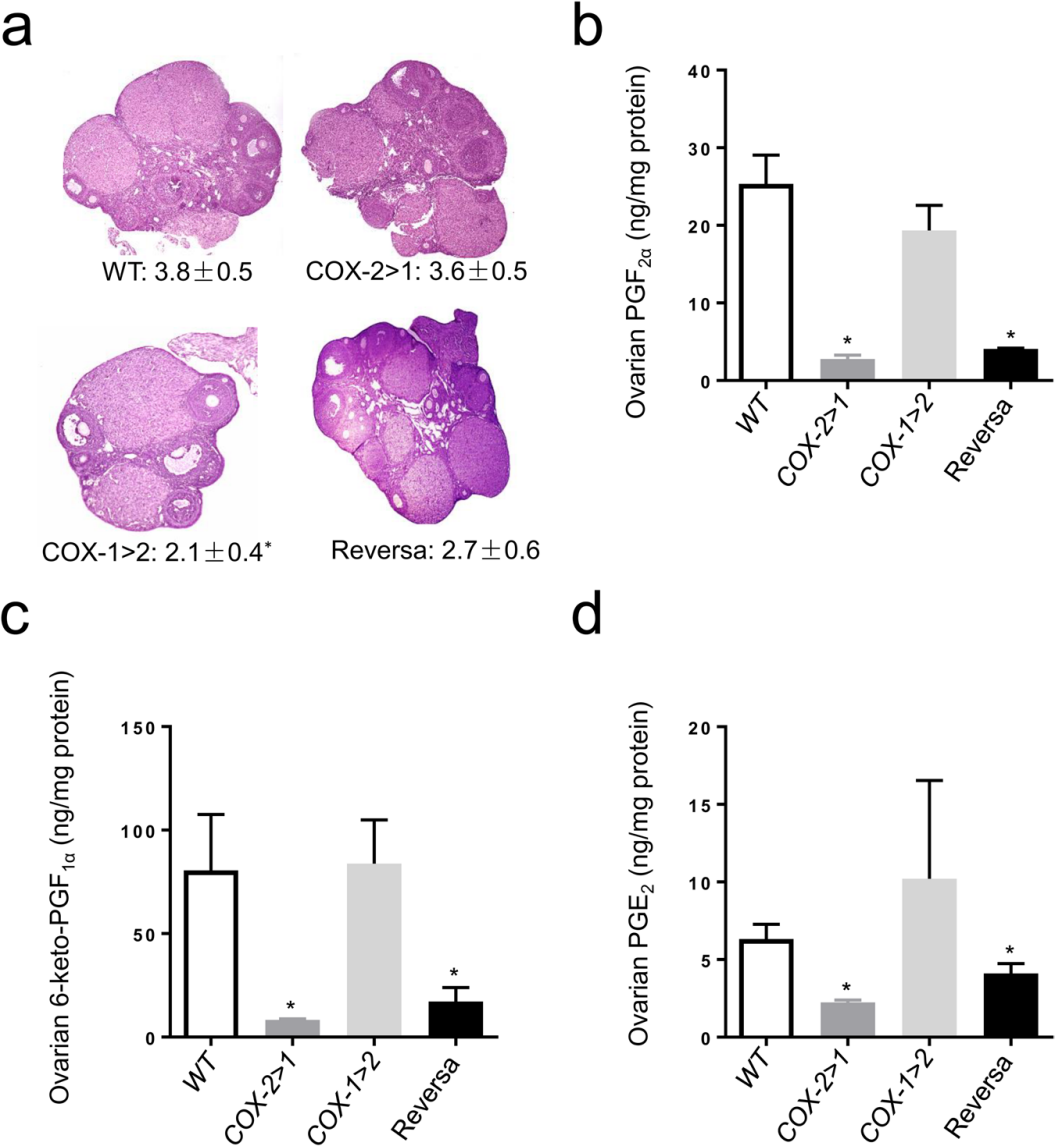
Ovarian histology and prostaglandin profile on gestation day 4.5. (**a**) Representative light photomicrographs (original magnification, × 50) of ovarian sections on gestation day 4.5. Numbers under each micrograph are means ± SEM of corpora lutea counted on sections. Data are presented as mean ± SEM, n = 5–7, ^*^P < 0.05 *vs* WT, one-way ANOVA analysis. (**b**), (**c**), and (**d**) Ovarian prostaglandin F_2α_ (PGF_2α_), 6-keto-prostaglandin F_1α_ (6-keto-PGF_1α_), and prostaglandin E_2_ (PGE_2_), respectively. Frozen ovaries (2–3 pooled) from the same group were subjected to prostaglandin extraction and measurement. Data are presented as mean ± SEM, n = 3, ^*^P < 0.05 *vs* WT, nonparametric tests.

In accordance with our previous findings, *Ptgs2*-deficiency in COX-1>COX-2 mice leads to lower levels of progesterone, and in turn impairs the formation of the corpus luteum. Ovarian morphology reveals that fewer mature corpora lutea form in COX-1>COX-2 mice on gestation day 4.5 (Fig. 4a). The apparent morphological appearance of these corpora lutea was normal with some showing signs of regression. Ovarian PG profiles are concordant with those in uterus, where COX-1 is the predominant source of all three PGs assayed (Fig. 4b–d). Surprisingly, successful transformation of corpora lutea does not seem to rely on these COX-1-derived PGs, but rather on the presence of COX-2, either in native form in WT mice or knock-in form in Reversa mice.

### High progesterone levels in Reversa mice delay the onset of parturition

**Figure 5:**
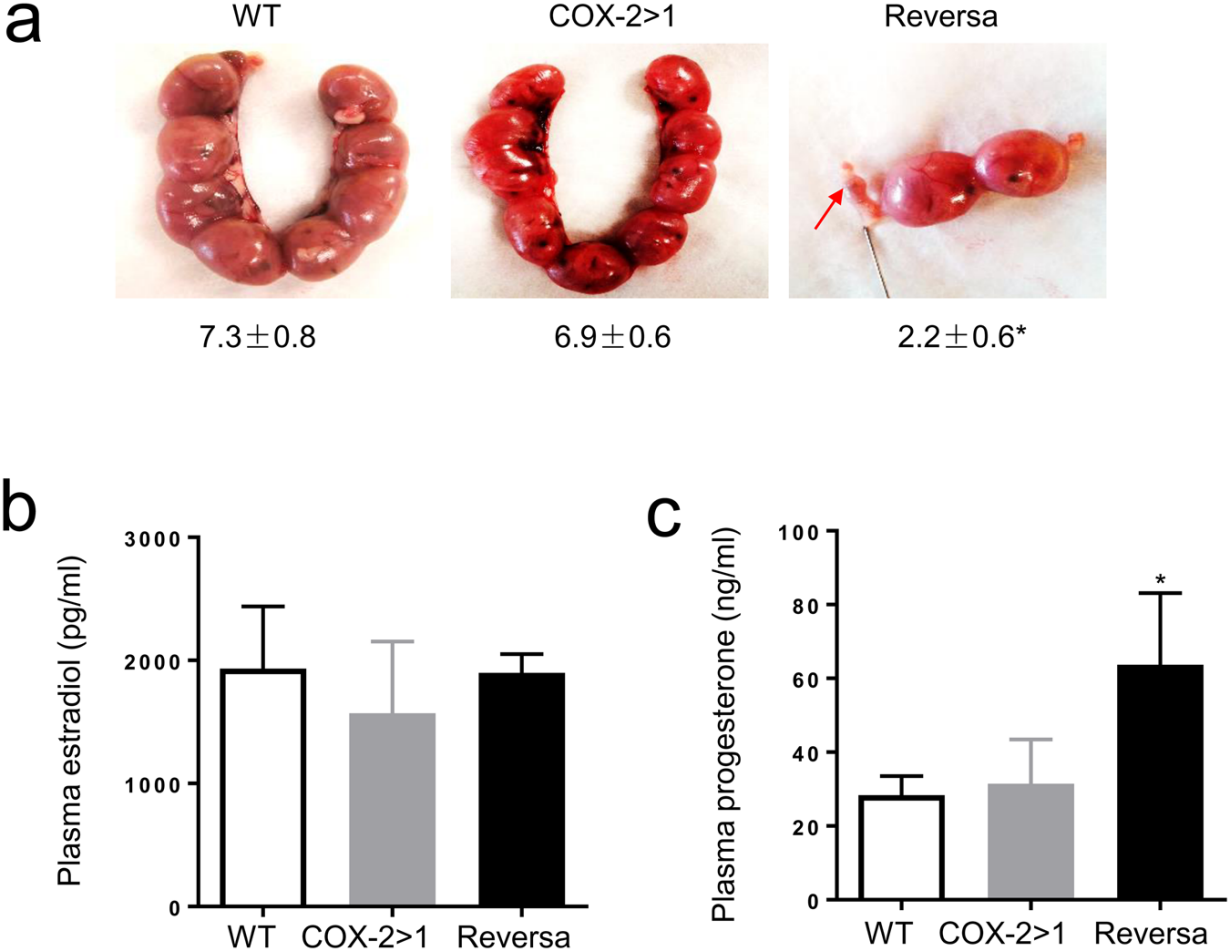
Fetal development and plasma sex hormones during late-stage pregnancy. (**a**) Representative uteri with fetuses from WT, COX-2>COX-1, and Reversa mice on gestation day 19 before the onset of parturition. These data were collected from female WT, COX-2>COX-1, and Reversa mice mating with males of the same genotype. No COX-1>COX-2 mice were pregnant in these experiments. A representative resorbed fetus (red arrow) was observed in the uterus of a Reversa mouse on d19 of pregnancy. Numbers under each gross specimen are means ± SEM of viable fetuses. Data are presented as mean ± SEM, n = 6–7, ^*^P < 0.05 *vs* WT, one-way ANOVA analysis. (**b**) Plasma estradiol and (**c**) progesterone concentration on gestation day 18–19. Blood was collected via submandibular vein from mice of each genotype (n = 5–6) and concentration was determined by sequential competitive immunoassay. Data are presented as mean ± SEM. ^*^P < 0.05 *vs* WT, one-way ANOVA analysis.

As mentioned earlier, Reversa mice have a markedly reduced litter size and a longer gestation length. Thus, we examined the effects of *Ptgs* gene exchange on late-stage pregnancy and onset of parturition. The number of viable fetuses in COX-2>COX-1 uteri was comparable with that of WT uteri on gestation day 19. In contrast, homozygous Reversa mice had fewer (n = 6, *P* < 0.05) and slightly smaller viable fetuses compared with WT mice (Fig. 5a). The resorbed fetuses could be identified at this stage, resulting in the smaller litter size in homozygous Reversa females (Table 2). This happened among implanted blastocysts during subsequent gestation, indicating that native COX-2 is crucial not only for proper implantation, but for embryo maturation and fetal development. We determined the plasma progesterone and estradiol levels in mice during late-stage pregnancy. As expected, estradiol levels did not differ among the three genotypes, as the placenta becomes their main source during pregnancy (Fig. 5b). Mice of each genotype demonstrated lower plasma progesterone levels during late-stage of pregnancy compared to those on d4.5, indicative of regressing activity of the corpus luteum. However, the progesterone levels in Reversa mice remained higher than in WT and COX-2>COX-1 mice (Fig. 5c), which resulted in delayed onset of parturition (Table 2).

### *Ptgs2* compensates for the loss of *Ptgs1* during late-stage pregnancy despite minimal capacity for PG biosynthesis

**Figure 6:**
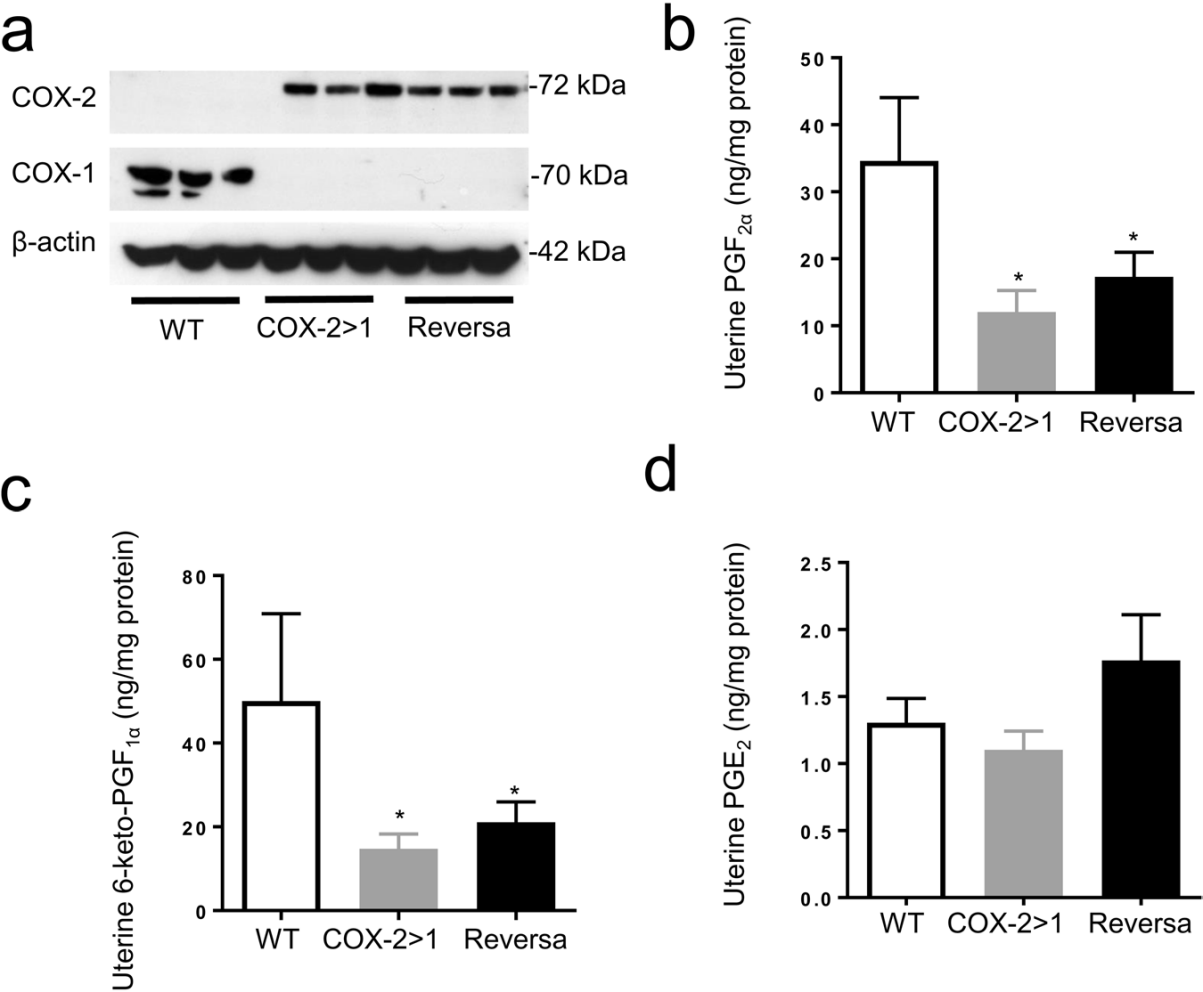
Uterine COX expression and prostanoid profiles in mice during late-stage pregnancy. (**a**) Western blot analysis of COX-1 and COX-2 expression in uteri on gestation day 19. (**b**), (**c**), and (**d**) Uterine prostaglandin F_2α_ (PGF_2α_), 6-keto-prstaglandin F_1α_ (6-keto-PGF_1α_), and prostaglandin E_2_ (PGE_2_), respectively. Prostaglandins were extracted from uteri isolated from each group and determined by competitive immunoassay, and further normalized with the protein level. Data are presented as mean ± SEM, n = 5–6, ^*^P < 0.05 *vs* WT, one-way ANOVA analysis.

To see whether COX-2 can offset the deficiency of COX-1, we first compared the expression of both isoforms during late-stage pregnancy (gestation day 19) in the uterus. COX-1, but not COX-2, is expressed in all WT uteri. As shown in Fig. 6a, with the targeting of *Ptgs* genes, COX-2 is expressed in the uteri of COX-2>COX-1 and Reversa mice in a pattern resembling the native expression of COX-1 in WT mice in both the non-gravid uterus and in the uterus of gestation d4.5 (Figs 1a and 3a). Strikingly, these knock-in COX-2 proteins are poor sources of PG formation: the capacity to form both PGF_2α_ and PGI_2_ in COX-2>COX-1 and Reversa mice is much lower than in WT mice (Fig. 6b and c). PGE_2_ levels are not significantly altered in COX-2>COX-1 and Reversa mice on day 19 of gestation (Fig. 6d), suggesting that PGE_2_, produced by both maternal and fetal tissues during pregnancy, may also play a role in the process of parturition^38^.

**Figure 7:**
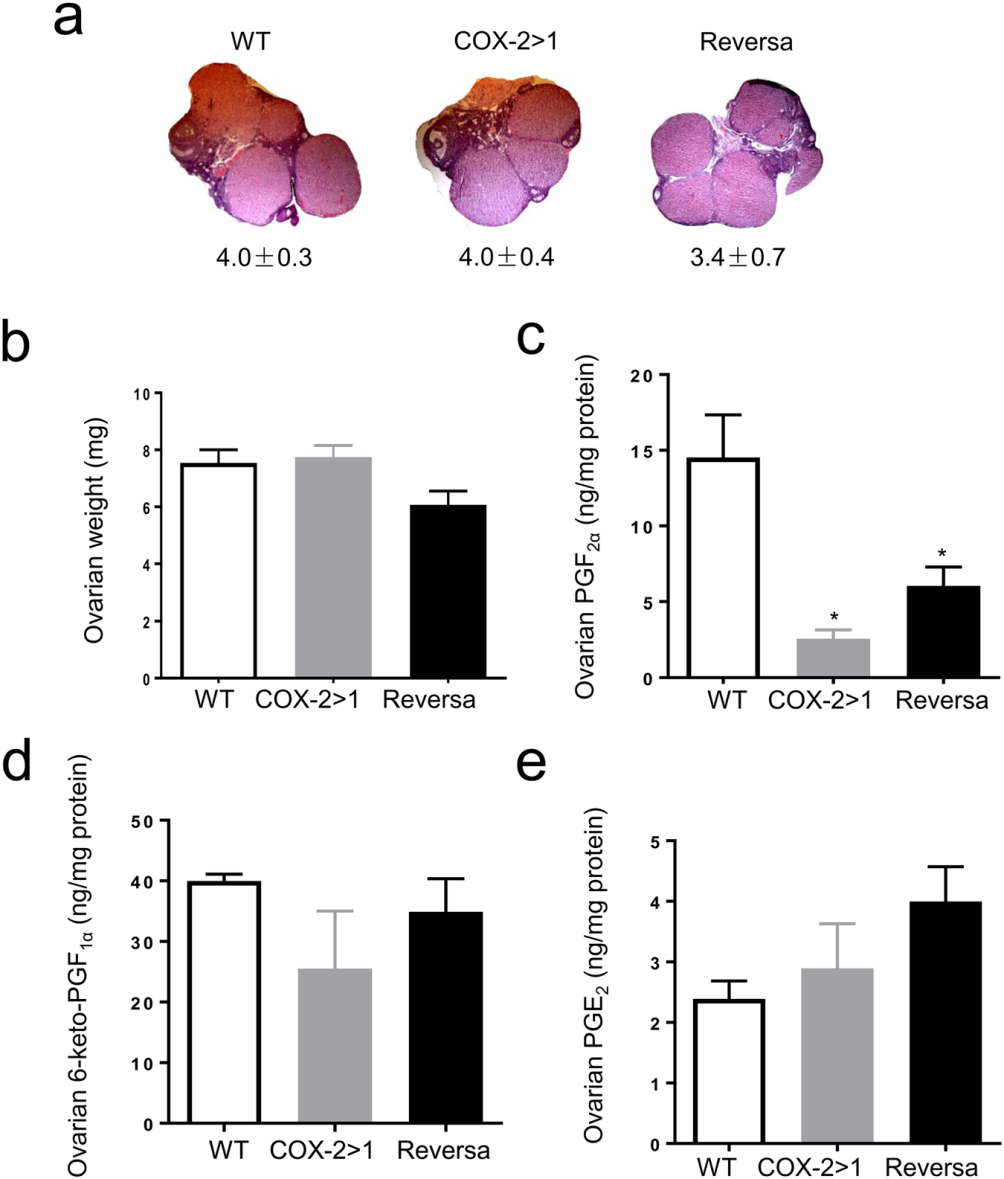
Corpus luteum histology and ovarian prostaglandin profiles on gestation day 19. (**a**) Representative H&E stained ovarian sections (original magnification, ×50) on gestation day 19. Numbers under each micrograph are means ± SEM of corpora lutea counted. Data are presented as mean ± SEM, n = 5–7. (**b**) Ovarian weights from pregnant mice on gestation day 19. Weights from both ovaries of each mouse were measured. Data are presented as mean ± SEM, n = 6–7. (**c**), (**d**) and (**e**) Ovarian prostaglandin F_2α_ (PGF_2α_), 6-keto-prostaglandin F_1α_ (6-keto-PGF_1α_), and prostaglandin E_2_ (PGE_2_), respectively. Frozen ovaries (2–3 pooled) were subjected to prostaglandin extraction prior to competitive immunoassay. n = 3, ^*^P < 0.05 *vs* WT, nonparametric tests.

We further investigated ovarian histology and PG profiles on day 19 of pregnancy. No remarkable morphological changes were observed in ovaries of COX-2>COX-1 and Reversa mice. The numbers of corpora lutea (Fig. 7a) and ovarian weights (Fig. 7b) were comparable with WT mice. Knock-in COX-2 in the ovaries of COX-2>COX-1 and Reversa mice was not able to compensate for PGF_2α_ (Fig. 7c), but appear to offset the loss of *Ptgs1* in the capacity of the ovary to generate PGE_2_ and 6-keto-PGF_1α_ at this stage of pregnancy (Fig. 7d and e). Taken together, *Ptgs2* is essential to compensate for the loss of *Ptgs1* during late-stage pregnancy, even though PG production remains deficient in COX-2>COX-1 mice, similar to our previous findings in COX-1 knockdown females^39^.

## Discussion

The present study provides new insights into the distinct roles of the two COX isoforms in implantation, parturition and fetal development. Normal female reproductive function appears to depend preferentially on COX-2. In particular, the novel genetic models (1) uncover COX-1 as the primary source of prostaglandins in the reproductive tract; (2) reveal that COX-2, but not COX-1, makes irreplaceable contributions to the implantation process; and (3) demonstrate the ability of constitutive COX-2 expression to compensate for loss of COX-1in parturition.

### Isoform-specific compensation in female reproduction

Pharmacological and genetic inhibition of the COX/PG pathways results in a compensatory isoform-specific response. COX-1 knock-down prevents platelet-dependent thrombosis but sustains parturition^39^. *Ptgs2* mRNA was strongly expressed in the uteri of COX-1-deficient mice on gestation days 1 and 8, which might partially compensate for the loss of COX-1 activity during implantation^36^. A compensatory up-regulation of COX-1 can occur in the uterus from mice missing *Ptgs2*. During early pregnancy, this compensatory up-regulation of *Ptgs1* functioned in a pattern similar to *Ptgs2* in initiating the attachment reaction in CD1 *Ptgs2* null mice. In contrast, this compensatory phenomenon was not observed in C57BL/6 J/129 *Ptgs2* null mice^30^. In the present study, although the COX-1 in COX-1>COX-2 mice has a comparable, if not enhanced capacity to generate PGs relative to WT mice, implantation defects could not be rescued. Insertion of *Ptgs1* under the *Ptgs2* regulatory sequences on a mixed C57BL/6 × 129 Sv genetic background partially rescues the impairment in fertility due to loss of COX-2^31^. In the current study, the disruption of the native COX-1 in COX-2>COX-1 and Reversa mice, which led to abnormal parturition^17,18^, can be preferentially compensated for by knock-in of COX-2 under the control of *Ptgs1* promoter on the C57BL/6 genetic background. In contrast, COX-2 disruption (COX-1>COX-2) displays severe reproductive impairment, which could be, at least partially, rescued by expression of knocked-in COX-2 in Reversa mice. Herein, most of COX-1’s role in implantation and late-stage pregnancy can be compensated for by COX-2, but not vice versa. COX-1>COX-2 mice had implantation sites, but could not sustain term labor. When and how the implanted blastocysts are lost in COX-1>COX-2 mice needs to be examined further.

### COX-1-derived prostaglandins in implantation and ensuing gestation

Our studies demonstrate that both uterine and ovarian COX-1 are the main source of PG synthesis on days 4.5 and 19 of pregnancy. Knockout of COX-1 dramatically reduces uterine PG levels on day 4 of pregnancy^18,36^, which is consistent with our current data. On the other hand, disruption of COX-2 in COX-1>COX-2 mice does not affect the PG levels assayed, which agrees with previous evidence that no significant difference in uterine PG profiles was noted between WT and COX-2-deficient mice on days 5 and 6 of pregnancy^30^. PGs alone, however, are not apparently sufficient to secure implantation and the ensuing gestation, although sustained levels of PGI_2_ appear to be important for implantation^37^, and PGF_2α_ for luteolysis and onset of parturition^23,40^ based on previous findings. Both uterine and ovarian PGE_2_ levels of COX-2>COX-1 mice are extremely low in our studies, despite evidence that PGE_2_ enhances decidualization in rats^41^. Our results are consistent with the inefficiency of exogenously administered PGE_2_ to induce optimal implantation^37^ and decidualization^16^ in COX-2-deficient mice. The failure of exogenous PGE_2_ in these respects might not be due to its unstable nature, rapid degradation, or ineffective delivery as supposed previously, but due to lack of COX-2 itself. Differences in the subcellular locations and functions of COX-1 and COX-2 may also be relevant. Both isoforms are anchored in the endoplasmic reticulum and nuclear membrane^5,42^, but it appears that COX-1 functions predominantly in the endoplasmic reticulum whereas COX-2 functions in the endoplasmic reticulum and the nuclear envelope^43^. We speculate that intranuclear generation of PGs from COX-2, despite very low levels, is perhaps the trigger for early implantation. Therefore, the difficulties in signaling of COX-1-derived PGs to the nuclear compartment could be one of the primary reasons for their limited effectiveness in securing the embryo implantation at day 4.5 of pregnancy. In addition, implantation and decidualization are not disturbed in F prostanoid (FP) receptor knockout mice^23^. Thus high levels of COX-1 generated PGF_2α_ synthesis do not appear crucial for these processes.

### Minimal PG biosynthetic capacity is involved in COX-2-dependent reproductive compensations

In contrast, highly expressed knock-in COX-2 (in either COX-2>COX-1 or Reversa mice) barely makes any PGs, but maintains all the aspects of female reproduction. One possibility is that the amount of PGs needed for normal reproductive functions is far less than the tissue capacity of COXs to generate them. In eicosanoid biology, the capacity of cells/tissues to make these lipid mediators strikingly exceeds their actual rates of biosynthesis^44^. For example, the discordance was reported previously in platelets, where the capacity to make TxA_2_ is 300–400 ng/ml as measured by TxB_2_ in serum, whereas maximal plasma concentration is ≈2 pg/ml^45^. Indeed, we previously determined that uterine and ovarian PGF_2α_ levels could be blocked by up to 92% in a COX-1 knockdown mouse model, and the residual PGs were still sufficient to promote timely luteolysis for initiation of labor^39^. This may explain why COX-1 deficient mice did not spontaneously develop gastric ulcers even though the gastric PGs levels in COX-1 null mice indicated a greater than 99% reduction^17^. These findings are also in agreement with previous studies showing the intact native COX-2 in COX-1 null mice, which generates PGs inefficiently^46^, maintains normal renal function^17^. Indeed, recently we have shown that the two COX isoforms can preferentially compensate for some renal function independent of the capacity to make PGs under basal conditions^34^. Collectively, despite a minimal capacity of reproductive tissues to make PGs, *Ptgs2* is sufficient to compensate for the loss of *Ptgs1* during implantation and late-stage pregnancy.

COX-2 is recognized to be critical for implantation, development of the corpus luteum and of the fetus. Difference in substrate selectivity of the two COX isoforms^2–4^ resulting in the ability of COX-2, but not COX-1, to metabolize the endocannabinoids arachidonoyl ethanolamide and 2-arachidonoyl glycerol, is proposed to be due to a slightly enlarged side pocket of the arachidonic acid-binding site in COX-2, which is absent in the COX-1 structure. Although endocannabinoids were not studied in the present studies, these mediators^47,48^ or yet unknown lipid mediators could direct aspects of early implantation and late-stage pregnancy.

Taken together, the delayed onset of parturition previously found in COX-1 null mice is rescued by *Ptgs2* exchange into the *Ptgs1* genetic locus in COX-2>COX-1 mice. However, knock-in COX-1 failed to substitute for the loss of COX-2. Thus, reproductive functions performed by COX-1 can be replaced by COX-2, but not vice versa. As in other tissues, a small residual capacity of COXs to generate PGs is sufficient to sustain these isoform-specific compensations in reproduction. An enhanced understanding of the pathophysiologic roles and mechanisms of PGs and other lipid mediators will benefit efforts to develop better therapeutic and diagnostic strategies in reproductive biology.

## Methods

### Mice

A COX-1>COX-2 mouse strain (JAX, 008104)^31^, on a mixed C57BL/6 × 129/Sv genetic background, was backcrossed with the C57BL/6 strain for 10 generations. A COX-2>COX-1 mouse strain, on the C57BL/6 background, was established as described previously^33^. Cross-breeding of COX-1>COX-2 and COX-2>COX-1 mice produces “Reversa” mice^32,33^. As shown in Table 1, the endogenous *Ptgs* alleles are still in the genome of a “single” knock-in mouse i.e. endogenous *Ptgs2* encoding the native COX-2 protein is in COX-2>COX-1 mice and endogenous *Ptgs1* encoding native COX-1 is present in COX-1>COX-2 mice, respectively. However, both native COX isoforms are disrupted and the *Ptgs1* and *Ptgs2* genes are inserted to permit them to substitute for one another in Reversa mice. Genotyping was performed routinely by PCR on DNA isolated from tail biopsies. All procedures for animal experimentation were undertaken in accordance with the principles and guidelines of the Canadian Council on Animal Care and were approved by the Queen’s University Animal Care Committee (approval #2012-029 and 2016-1673).

### Estrous cycle

To evaluate the estrous cycle of female mice (10–12 weeks old), vaginal lavage was performed daily at 10 am for 12 consecutive days using 100 µl of autoclaved distilled water. The fluid was then transferred to a microscope slide and left to dry. The slides were stained with 0.1% Crystal Violet and sealed with Cytoseal 60. The vaginal smears were analyzed under a microscope (Leica, DM IRB, Richmond Hill, ON). Identification of relative numbers of nucleated epithelial cells, cornified epithelial cells, and leukocytes determined the estrous stage, as previously reported^49^. Two cycles were generally captured for each mouse and then averaged to obtain cycle length for each mouse.

### Implantation sites

Fertile WT males on a C57BL/6 genetic background were mated to female COX-1>COX-2, COX-2>COX-1 and Reversa mice (10–12 weeks old). Gestation day 0.5 (d0.5) was established to be the day of the first sighting of vaginal plugs in female mice. Implantation sites in the uterus were visualized using Evan’s blue dye (1%, 0.1 ml) through tail vein injection on gestation day 4.5 (d4.5), and allowed to circulate for 5 minutes. The mice were subsequently euthanized by CO_2_ inhalation and their uteri were dissected and implantation sites were counted. Pregnancy was confirmed via blue-stained sites and the percentage was calculated (positive pregnancy % = mice that had positively stained sites/mice that were positively plugged).

### Gestation period and litter size

The significance of COX substitution on female reproductive function was further assessed through gestation period and litter size. In the first set of experiments, these data were collected from female WT, COX-1>COX-2, COX-2>COX-1, and Reversa mice mating with males of the same genotype, or with fertile WT males, respectively. Because female COX-2-deficient mice display multiple reproductive defects, we failed to generate any COX-1>COX-2 mice from numerous homozygous parent matings. In the second set of experiments, heterozygous COX-1>COX-2 females were paired with homozygous COX-1>COX-2 males; Female Reversa mice with two copies of *Ptgs2*>*Ptgs1* (homozygous), but one copy of *Ptgs1*>*Ptgs2* (heterozygous) were paired with homozygous Reversa males. The litter size only includes live pups within 24 h after birth.

### Ovarian weights and histology

The weights of ovaries from 10–12-week-old mice on gestation day 19.5 (d19.5), or from non-gravid mice, were determined as described previously^39^. Dissected ovaries were fixed in 10% buffered-formalin for 24 h, processed routinely, and then embedded in paraffin for staining with hematoxylin and eosin (H&E). Whole ovary section pictures (×50) were captured with a microscope (Leica, DM IRB, Richmond Hill, ON) and numbers of corpora lutea were counted in 5 random sections from each ovary. All specimens were analyzed by an investigator blinded to the study design.

### Blood sex hormone assay

Blood was drawn with heparin-coated capillary tubes from the submandibular vein on d 0.5 when a plug was found and again on d 4.5 (for implantation site experiments) and d 18.5–19.5 (for gestation period and litter size experiments). The plasma was saved for measurement of estradiol and progesterone at different time points of pregnancy, using enzyme immunoassay (EIA) kits (Cayman Chemical, Ann Arbor, MI).

### Enzyme immunoassay of PGs

The measurements of PGE_2_, PGF_2α_, and 6-keto-PGF_1α_ (the stable hydrolysis product of PGI_2_) levels were carried out in the homogenates of uterine or ovarian tissues, as previously described^50^ with some modifications. Briefly, dissected uteri were weighed and rapidly homogenized in 4 volumes (wt/vol) of ice cold PBS containing 100 μM indomethacin followed by ultrasonification. Residual tissue was separated by centrifugation, and the supernatant was collected, then assayed (Bradford method) to determine protein concentration. The homogenates were acidified to pH3.0 with 1 M HCl and extracted for PG determination three times with 1 volume of ethyl acetate. Pooled ethyl acetate extracts were dried under N_2_ and reconstituted with 100 µl buffer and PGE_2_, PGF_2α_, and 6-keto-PGF_1α_ were determined by competitive enzyme immunoassay (Cayman Chemical, Ann Arbor, MI). Data were normalized to the protein content in the tissue preparation. Ovarian samples were processed the same way except that 2–3 frozen ovaries from the same group were pooled prior to PG extraction.

### Western blot analysis

Uterine lysates were prepared with T-PER protein extraction reagent (Thermo Scientific) with a protease inhibitor cocktail (Roche Diagnostics, Indianapolis, IN). Western blot analysis was carried out as outlined previously^33^. Original images of Western blots are presented in the Electronic supplementary material.

### Data analysis

Data are expressed as mean ± SEM. The Kolmogorov-Smirnov test was used as a normality test. One-way ANOVA analysis was used for normally distributed variables, and Dunnett T3 *post hoc* comparisons were conducted when the ANOVA indicated a significant difference among the compared means. Nonparametric Kruskal-Wallis tests were used for non-normally distributed variables. Tests were 2-tailed, and values of *P* < 0.05 were considered statistically significant. A *Chi*-square test was performed to determine if there were significant differences between the percentage of positive pregnancies between groups. The statistical analysis was performed by GraphPad Prism 7 software (GraphPad, San Diego, CA).

## Electronic supplementary material

Supplementary figures

## Data Availability

All data generated or analyzed during this study are included in this published article (and its Supplementary Information files).

## References

[1] Smith WL, Urade Y, Jakobsson PJ (2011). Enzymes of the cyclooxygenase pathways of prostanoid biosynthesis. Chem Rev.

[2] Yu M, Ives D, Ramesha CS (1997). Synthesis of prostaglandin E2 ethanolamide from anandamide by cyclooxygenase-2. J Biol Chem.

[3] Kozak KR, Prusakiewicz JJ, Rowlinson SW, Prudhomme DR, Marnett LJ (2003). Amino acid determinants in cyclooxygenase-2 oxygenation of the endocannabinoid anandamide. Biochemistry.

[4] Kozak KR, Prusakiewicz JJ, Marnett LJ (2004). Oxidative metabolism of endocannabinoids by COX-2. Curr Pharm Des.

[5] Spencer AG, Woods JW, Arakawa T, Singer II, Smith WL (1998). Subcellular localization of prostaglandin endoperoxide H synthases-1 and -2 by immunoelectron microscopy. J Biol Chem.

[6] Yuan C, Smith WL (2015). A cyclooxygenase-2-dependent prostaglandin E2 biosynthetic system in the Golgi apparatus. J Biol Chem.

[7] Vane JR, Bakhle YS, Botting RM (1998). Cyclooxygenases 1 and 2. Annu Rev Pharmacol Toxicol.

[8] Funk CD (2001). Prostaglandins and leukotrienes: advances in eicosanoid biology. Science.

[9] Narumiya S, FitzGerald GA (2001). Genetic and pharmacological analysis of prostanoid receptor function. J Clin Invest.

[10] Stavreus-Evers A, Koraen L, Scott JE, Zhang P, Westlund P (2005). Distribution of cyclooxygenase-1, cyclooxygenase-2, and cytosolic phospholipase A2 in the luteal phase human endometrium and ovary. Fertil Steril.

[11] Chakraborty I, Das SK, Wang J, Dey SK (1996). Developmental expression of the cyclo-oxygenase-1 and cyclo-oxygenase-2 genes in the peri-implantation mouse uterus and their differential regulation by the blastocyst and ovarian steroids. J Mol Endocrinol.

[12] Sirois J, Simmons DL, Richards JS (1992). Hormonal regulation of messenger ribonucleic acid encoding a novel isoform of prostaglandin endoperoxide H synthase in rat preovulatory follicles. Induction in vivo and in vitro. J Biol Chem.

[13] Sirois J (1994). Induction of prostaglandin endoperoxide synthase-2 by human chorionic gonadotropin in bovine preovulatory follicles *in vivo*. Endocrinology.

[14] Song JH, Sirois J, Houde A, Murphy BD (1998). Cloning, developmental expression, and immunohistochemistry of cyclooxygenase 2 in the endometrium during embryo implantation and gestation in the mink (Mustela vison). Endocrinology.

[15] Dinchuk JE (1995). Renal abnormalities and an altered inflammatory response in mice lacking cyclooxygenase II. Nature.

[16] Lim H (1997). Multiple female reproductive failures in cyclooxygenase 2-deficient mice. Cell.

[17] Langenbach R (1995). Prostaglandin synthase 1 gene disruption in mice reduces arachidonic acid-induced inflammation and indomethacin-induced gastric ulceration. Cell.

[18] Reese J (2000). Coordinated regulation of fetal and maternal prostaglandins directs successful birth and postnatal adaptation in the mouse. Proc Natl Acad Sci USA.

[19] Bany, B. M. & Kennedy, T. G. Regulation of cyclooxygenase gene expression in rat endometrial stromal cells: the role of epidermal growth factor. *Dev Genet* **21**, 109–115, 10.1002/(SICI)1520-6408 (1997). 10.1002/(SICI)1520-6408(1997)21:1<109::AID-DVG13>3.0.CO;2-5 9291587

[20] Davis BJ (1999). Anovulation in cyclooxygenase-2-deficient mice is restored by prostaglandin E2 and interleukin-1beta. Endocrinology.

[21] Brown CG, Poyser NL (1984). Studies on ovarian prostaglandin production in relation to ovulation in the rat. J Reprod Fertil.

[22] Hizaki H (1999). Abortive expansion of the cumulus and impaired fertility in mice lacking the prostaglandin E receptor subtype EP(2). Proc Natl Acad Sci USA.

[23] Sugimoto Y (1997). Failure of parturition in mice lacking the prostaglandin F receptor. Science.

[24] Powell WS, Hammarstrom S, Samuelsson B, Sjoberg B (1974). Letter: Prostaglandin-F2alpha receptor in human corpora lutea. Lancet.

[25] Bennegard B, Hahlin M, Wennberg E, Noren H (1991). Local luteolytic effect of prostaglandin F2 alpha in the human corpus luteum. Fertil Steril.

[26] Matsumoto H (2002). Cyclooxygenase-2 differentially directs uterine angiogenesis during implantation in mice. J Biol Chem.

[27] Ruan YC (2012). Activation of the epithelial Na+channel triggers prostaglandin E(2) release and production required for embryo implantation. Nat Med.

[28] Waclawik A, Kaczynski P, Jabbour HN (2013). Autocrine and paracrine mechanisms of prostaglandin E(2) action on trophoblast/conceptus cells through the prostaglandin E(2) receptor (PTGER2) during implantation. Endocrinology.

[29] Cheng JG, Stewart CL (2003). Loss of cyclooxygenase-2 retards decidual growth but does not inhibit embryo implantation or development to term. Biol Reprod.

[30] Wang H (2004). Rescue of female infertility from the loss of cyclooxygenase-2 by compensatory up-regulation of cyclooxygenase-1 is a function of genetic makeup. J Biol Chem.

[31] Yu Y (2007). Targeted cyclooxygenase gene (ptgs) exchange reveals discriminant isoform functionality. J Biol Chem.

[32] Li X (2018). Genomic and lipidomic analyses differentiate the compensatory roles of two COX isoforms during systemic inflammation in mice. J Lipid Res.

[33] Li X (2018). Flipping the cyclooxygenase (Ptgs) genes reveals isoform-specific compensatory functions. J Lipid Res.

[34] Li, X. *et al*. Differential compensation of two cyclooxygenases in renal homeostasis is independent of prostaglandin-synthetic capacity under basal conditions. *FASEB J, fj201800252R*, 10.1096/fj.201800252R (2018). 10.1096/fj.201800252R PMC6133703 29676940

[35] Das SK (1994). Heparin-binding EGF-like growth factor gene is induced in the mouse uterus temporally by the blastocyst solely at the site of its apposition: a possible ligand for interaction with blastocyst EGF-receptor in implantation. Development.

[36] Reese J, Brown N, Paria BC, Morrow J, Dey SK (1999). COX-2 compensation in the uterus of COX-1 deficient mice during the pre-implantation period. Mol Cell Endocrinol.

[37] Lim H (1999). Cyclo-oxygenase-2-derived prostacyclin mediates embryo implantation in the mouse via PPARdelta. Genes Dev.

[38] Olson DM, Ammann C (2007). Role of the prostaglandins in labour and prostaglandin receptor inhibitors in the prevention of preterm labour. Front Biosci.

[39] Yu Y (2005). Differential impact of prostaglandin H synthase 1 knockdown on platelets and parturition. J Clin Invest.

[40] Horton EW, Poyser NL (1976). Uterine luteolytic hormone: a physiological role for prostaglandin F2alpha. Physiol Rev.

[41] Kennedy TG (1985). Evidence for the involvement of prostaglandins throughout the decidual cell reaction in the rat. Biol Reprod.

[42] Otto JC, Smith WL (1994). The orientation of prostaglandin endoperoxide synthases-1 and -2 in the endoplasmic reticulum. J Biol Chem.

[43] Morita I (1995). Different intracellular locations for prostaglandin endoperoxide H synthase-1 and -2. J Biol Chem.

[44] Grosser T, Naji A, FitzGerald GA (2018). Urinary Prostaglandin Metabolites: An Incomplete Reckoning and a Flush to Judgment. Circ Res.

[45] Patrono C (1986). Estimated rate of thromboxane secretion into the circulation of normal humans. J Clin Invest.

[46] Qi Z, Cai H, Morrow JD, Breyer MD (2006). Differentiation of cyclooxygenase 1- and 2-derived prostanoids in mouse kidney and aorta. Hypertension.

[47] Wang H (2004). Aberrant cannabinoid signaling impairs oviductal transport of embryos. Nat Med.

[48] Sun X (2010). Endocannabinoid signaling directs differentiation of trophoblast cell lineages and placentation. Proc Natl Acad Sci USA.

[49] Byers SL, Wiles MV, Dunn SL, Taft RA (2012). Mouse estrous cycle identification tool and images. PLoS One.

[50] Sander VA, Piehl L, Facorro GB, Rubin de Celis E, Motta AB (2008). Regulation of functional and regressing stages of corpus luteum development in mice. Role of reactive oxygen species. Reprod Fertil Dev.

