# Supplementary material for: Isoform-Specific Compensation of Cyclooxygenase (Ptgs) Genes during Implantation and Late-Stage Pregnancy
Source: Sci Rep. 2018 Aug 14;8:12097. doi: 10.1038/s41598-018-30636-x (PMC6092371; doi:10.1038/s41598-018-30636-x)
Supplement: Supplementary file 1 — Supplementary figures [file 41598_2018_30636_MOESM1_ESM.pdf]

# Isoform-Specific Compensation of Cyclooxygenase (*Ptgs*) Genes during Implantation and Late-Stage Pregnancy

Xinzhi Li[1], Laurel L. Ballantyne[1], Mackenzie C. Crawford[1], Garret A. FitzGerald[2], Colin D. Funk[1*]

[1]Department of Biomedical and Molecular Sciences, Queen’s University, Kingston, ON Canada

[2]Institute for Translational Medicine and Therapeutics, Perelman School of Medicine, University of Pennsylvania, Philadelphia, PA, USA

[*]*Correspondence should be addressed to Colin D. Funk, Department of Biomedical and Molecular Sciences, 433 Botterell Hall, 18 Stuart Street, Queen's University, Kingston, Ontario, K7L 3N6, Canada. Email: funkc@queensu.ca*

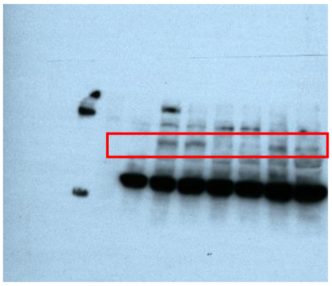
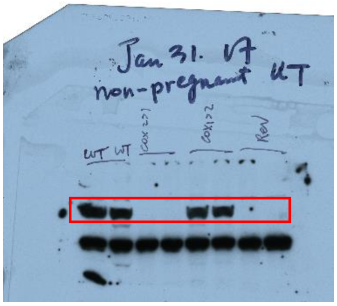

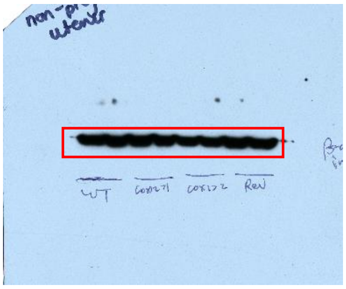

Figure 1. a COX-2 COX-1 β-actin

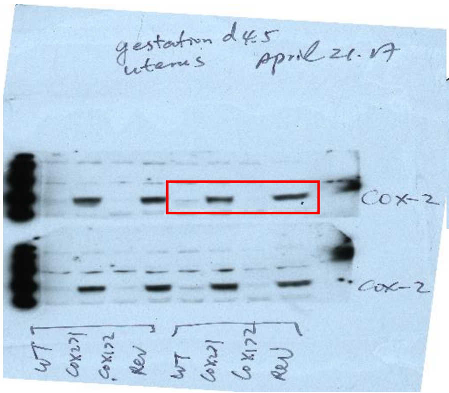

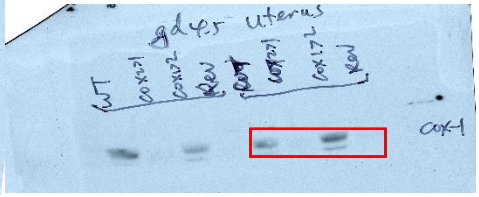

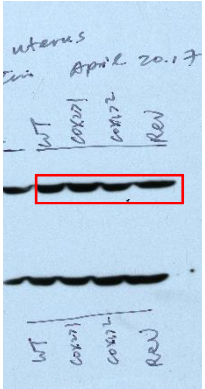


Figure 3. a COX-2 COX-1 β-actin

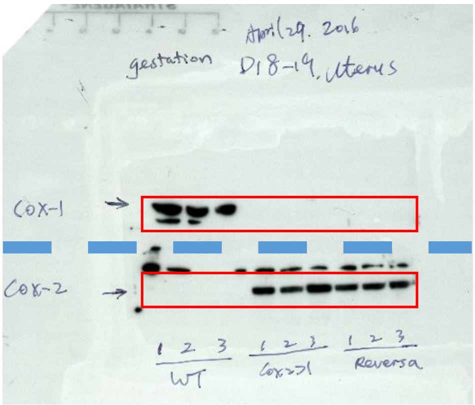

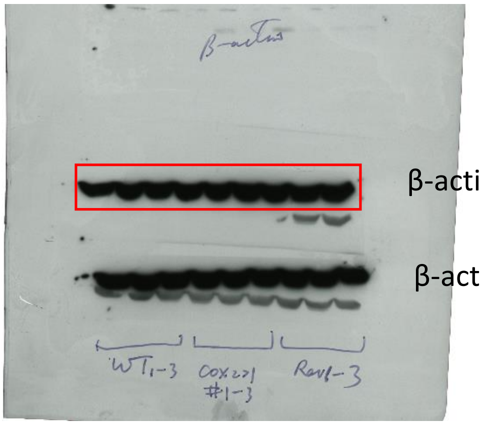


Figure 6. a COX-2 and COX-1. This picture was taken from two blots, upper part was probed with COX-1 antibody, lower part was probed with COX-2 antibody.

β-actin

**Supplementary figures**: Images of western blots presented in the main manuscript as Figure 1a, Figure 3a and Figure 6a. Sometimes the membranes were cut immediately after transfer to incubate in different primary antibodies, which were then probed individually for the proteins of interest, as appropriate for their molecular weights.
